# Fabrication of Carboxymethylcellulose/Metal-Organic Framework Beads for Removal of Pb(II) from Aqueous Solution

**DOI:** 10.3390/ma12060942

**Published:** 2019-03-21

**Authors:** Huo-Xi Jin, Hong Ping Xu, Nan Wang, Li-Ye Yang, Yang-Guang Wang, Di Yu, Xiao-Kun Ouyang

**Affiliations:** School of Food and Pharmacy, Zhejiang Ocean University, Zhoushan 316022, China; jinhuoxi@163.com (H.-X.J.); xhp3201@sina.com (H.P.X.); ynwangnan@163.com (N.W.); liyey@zjou.edu.cn (L.-Y.Y.); ygw0510@sohu.com (Y.-G.W.); oyxk1973@163.com (D.Y.)

**Keywords:** carboxymethylcellulose, adsorption, Pb(II), MOF-5

## Abstract

The ability to remove toxic heavy metals, such as Pb(II), from the environment is an important objective from both human-health and ecological perspectives. Herein, we describe the fabrication of a novel carboxymethylcellulose-coated metal organic material (MOF-5–CMC) adsorbent that removed lead ions from aqueous solutions. The adsorption material was characterized by Fourier-transform infrared spectroscopy, X-ray diffractometry, scanning electron microscopy, and X-ray photoelectron spectroscopy. We studied the functions of the contact time, pH, the original concentration of the Pb(II) solution, and adsorption temperature on adsorption capacity. MOF-5–CMC beads exhibit good adsorption performance; the maximum adsorption capacity obtained from the Langmuir isotherm-model is 322.58 mg/g, and the adsorption equilibrium was reached in 120 min at a concentration of 300 mg/L. The adsorption kinetics is well described by pseudo-second-order kinetics, and the adsorption equilibrium data are well fitted to the Langmuir isotherm model (R^2^ = 0.988). Thermodynamics experiments indicate that the adsorption process is both spontaneous and endothermic. In addition, the adsorbent is reusable. We conclude that MOF-5–CMC is a good adsorbent that can be used to remove Pb(II) from aqueous solutions.

## 1. Introduction

Owing to increasing global industrialization, heavy-metal pollution has become an important issue that can no longer be ignored [1]; heavy metals seriously threaten both environment and human health [2]. Lead(II) is a toxic heavy metal that acutely poisons nerves, blood, the digestive system, blood vessels, and kidneys when certain concentrations are reached, leading to multiple systemic organ diseases [3,4,5]. Moreover, the lack of degradability exhibited by Pb(II) leads to its continuous enrichment in water [5,6], which has resulted in irreversible harm to the environment, as well as humans once it enters the body. Therefore, the removal of Pb(II) is imperative [7,8].

Because of the availability of diverse metal centers and organic ligands, metal-organic frameworks (MOFs) have rapidly developed as porous materials in recent years [9]. A MOF is a multidimensional network-structured material formed by coordination hybridization of multidentate organic ligands and transition-metal ions under specific conditions [10,11]. Among them, MOF-5 is a type of multifunctional MOF nanomaterial that is porous and more mature than some other MOFs [12]. MOF-5 is made from terephthalic acid (BDC) and zinc nitrate (Zn(NO_3_)_2_) dissolved in *N*,*N*-dimethylacetamide (DMF) [13,14,15]. MOF-5 has a hollow skeletal structure and a high specific surface area [16]; therefore, it has good application prospects for the development of new materials, such as adsorbents and drug-loading materials [17,18,19].

Currently, environmentally friendly high-molecular-weight carbohydrate polymers are attracting worldwide attention [20,21]. Carboxymethylcellulose (CMC) is a water-soluble anionic cellulose ether; its diverse physical and chemical properties [22] have led to its wide-ranging applications in food, medicine, and wastewater treatment [23,24]. CMC exhibits excellent compatibility with water and can form hydrogels; it is resistant to high temperatures, its structure is stable at pH 2–11, and it shows good biodegradability and affinity [25]. CMC hydrogels exhibit good adsorption capacities, making them ideal materials for removing contaminants from aqueous solutions. CMC is an anionic polysaccharide that contains abundant carboxyl groups; consequently, it can adsorb heavy metals in a facile manner [26,27,28]. Based on these considerations, the excellent role that CMC plays in removing heavy-metal ions cannot be ignored.

Presently, capacitive-deionization and microbial-treatment technologies, as well as precipitation and adsorption methods, for the removal of lead ions from aqueous solutions, have been reported [29,30]. The adsorption method is considered extremely convenient owing to its efficiency and cost effectiveness. This study employed carboxymethylcellulose-coated MOF-5, which combines the complexing advantages of the carboxymethyl group toward metal ions and the excellent adsorption performance of MOF-5, to eliminate Pb(II) from aqueous solutions. In addition, MOF-5/CMC mixtures form beads in AlCl_3_ solution; these MOF-5–CMC beads are easily separated from other materials.

This study combines the advantages of CMC and MOF-5 materials. New adsorption beads were prepared by the CMC coating of MOF-5, which resulted in a material with a much larger adsorbing ability than either of the original two materials; it is also easy to recycle and reuse. The new adsorbent was comprehensively characterized, and the effects of adsorption temperature, initial concentration, adsorption time, pH, and the amount of adsorbent on the adsorbing ability of the novel material were explored. The data were adapted to an adsorption-isotherm equation and the adsorption thermodynamics was studied for the purpose of providing insight into the adsorption mechanism of the beads and to produce a new type of readily prepared adsorbent material with excellent adsorption performance that can be recycled and separated.

## 2. Materials and Methods 

### 2.1. Materials

Zinc nitrate hexahydrate (Zn(NO_3_)_2_·6H_2_O), terephthalic acid (BDC), and carboxymethylcellulose (CMC) were obtained from the Aladdin Reagent Co., Ltd. (Shanghai, China); *N*,*N*-dimethylacetamide (DMF), methanol (CH_3_OH), and aluminum chloride (AlCl_3_) were obtained from the Sinopharm Chemical Reagent Co., Ltd. (Shanghai, China).

### 2.2. Material Preparation and Characterization

#### 2.2.1. Preparation of MOF-5

The previously reported method was slightly modified to prepare MOF-5 [31]. Zn (NO_3_)_2_·6H_2_O (7.72 g) and BDC (1.64 g) were first dissolved in 225.6 mL of DMF. The mixture was then transferred into a reaction vessel where the reaction was run at 130 °C for 24 h. After waiting for the reaction temperature to reach room temperature, the crystals were collected by filtration and washed five times with DMF to remove unreacted zinc ions and BDC, after which it was washed five times with methanol to remove DMF. The sample was dried at 120 °C following the complete evaporation of methanol. 

#### 2.2.2. Synthesis of MOF-5–CMC Beads

CMC (0.9 g) was solubilized in 15 mL of distilled water. Concurrently, MOF-5 (0.9 g) was also dispersed in 15 mL of distilled water. The two liquids were mixed, and the resulting solution was slowly dripped by syringe into a solution of 2.5% aqueous AlCl_3_ and allowed to stand for 4 h to facilitate bead solidification. The beads were washed five times with deionized water to remove AlCl_3_. The preparation of CMC beads was performed with the same method with MOF-5–CMC or without MOF-5. 

#### 2.2.3. Characterization

Fourier transform infrared spectroscopy (FT-IR) was performed using a Thermo Nicolet 6700 spectrometer (Shimadzu, Tokyo, Japan) and KBr particles. X-ray photoelectron spectroscopy (XPS) was performed using an AXIS ULTRA DLD spectrometer (Kratos, Manchester, England). Scanning electron microscopy (SEM) was performed on a Hitachi S-4800 SEM instrument (Hitachi, Tokyo, Japan). X-ray diffraction (XRD, D8 Advance, Bruker, Karlsruhe, Germany) was used to ascertain material compositions. Thermogravimetric analysis (TGA, Q500, TA Instruments, New Castle, USA) was used to determine the stabilities of the materials at various temperatures.

#### 2.2.4. Adsorption Experiments

Pb(NO_3_)_2_ (1.6 g) was accurately weighed and added to 1000 mL of distilled water. Solutions with varying lead-ion concentrations (10–1000 mg/L) were prepared by diluting this solution. The pH was controlled to be in the 2–6 range through the addition of 0.1 M NaOH or 0.1 M HCl. The adsorption of Pb(II) was carried out in a shaker at 150 rpm. Atomic absorption spectrophotometry (AAS, AA-7700 Shinada) was applied to determine the Pb(II) contents remaining in the solution after Pb(II)-adsorption equilibrium. The equilibrium Pb(II)-adsorption capacity was calculated by applying the following formula:

(1) $$q_{e}=\frac{\left(C_{0}-C_{e}\right)V}{m}$$

*C_0_* (mg/L) is the original concentration of Pb(II), *C_e_* (mg/L) is the concentration of Pb(II) remaining in solution at equilibrium, *V* (L) is the volume of the Pb(II) solution, and *m* (g) is the weight of the MOF-5–CMC beads.

The adsorption capacity at a specific time was calculated by applying the following formula:

(2) $$q_{t}=\frac{\left(C_{0}-C_{t}\right)V}{m}$$

*C_t_* (mg/L) is the concentration of Pb(II) remaining in solution at *t*, and the remaining symbols are as defined above; *q_t_* (mg/g) is the adsorption capacity of MOF-5–CMC at specific time *t*.

The Pb(II) removal rate was calculated using the following formula:

(3) $$R=\frac{C_{0}-C_{e}}{C_{0}}\times 100\%$$

*R*(%) is the removal rate of Pb(II) by MOF-5–CMC.

#### 2.2.5. Reuse Experiments

A 0.1 M HCl solution was applied to desorb the Pb(II) adsorbed onto the MOF-5–CMC. At room temperature, the MOF-5–CMC–Pb(II) beads and 20 mL of 0.1 M HCl solution were shaken in a 100 mL shaker jar at 150 rpm for 4 h. Pb(II) was not detected in the eluent solution after the above steps were repeated four times. The MOF-5–CMC reused four times for adsorbing Pb(II), and its adsorption capacity was ascertained after each use.

## 3. Results and Discussion

### 3.1. Morphologies as Observed by SEM

**Figure 1:**
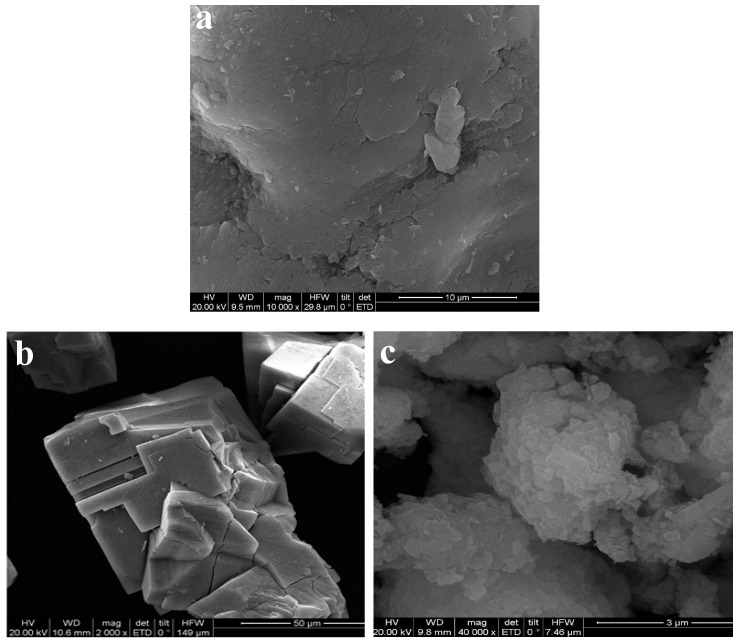
Scanning electron microscopy (SEM) images of (**a**) Carboxymethylcellulose (CMC), (**b**) MOF-5, and (**c**) MOF-5–CMC.

SEM images of CMC, MOF-5, and MOF-5–CMC are displayed in Figure 1, which reveals that MOF-5 has smooth surfaces and is highly crystalline and regular in shape [18]. The surface morphology of CMC is very rough, and many small particles are compactly gathered together, resulting in a large contact area that is beneficial for heavy metal ion adsorption [32,33]. The CMC-coated MOF-5 (MOF-5–CMC, Figure 1c) exhibits a rough and irregular morphology, which is attributable to hydrogen bonding involving the carboxyl and hydroxyl functional groups [34]; rough surfaces promote the adsorption of Pb(II). 

### 3.2. Thermogravimetric Analyses

**Figure 2:**
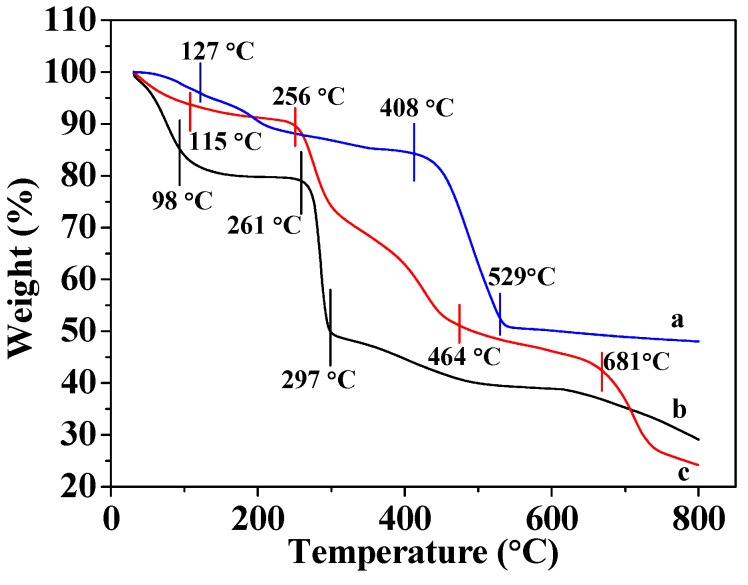
Thermogravimetric analysis (TGA) traces for (a) MOF-5, (b) MOF-5–CMC, and (c) CMC (The TGA conditions were gas flow 100 mL/min; atmosphere, air; temperature ramp, 10 °C/min.)

Figure 2c shows that CMC gradually loses mass in the 31–98 °C range due to losing water; the mass loss range of 98 to 297 °C is due to the decomposition of organic matter into inorganic matter, and is consistent with literature descriptions [32,35]. Figure 2a reveals that the mass of MOF-5 gradually decreases as it is heated from 31 to 127 °C, owing to the volatilization of hydrone. The weight loss is observed in the 127–408 °C range, which is ascribable to the thermal evaporation of organic ligand molecules, and the rapid exothermic process observed between 408 and 529 °C is consistent with the decomposition of MOF-5 into inorganic matter. The weight of MOF-5 did not decrease further at temperatures above 500 °C, indicating that only inorganic matter remained after decomposition. The results are identical to those described in the literature [12,18]. Figure 2b reveals that MOF-5–CMC undergoes more severe losses weight, indicating that this adsorbent is less thermally stable than MOF-5. The slight loss in weight in the 31–115 °C range is attributed to volatilization of water molecules and the loss of weight observed between 256 and 464 °C is due to the cleavage of the glycosidic bonds in CMC and the loss of carbonyl and carboxyl groups during the formation of relatively low-molecular-weight volatile compounds [23,34,36]. At the same time, the organic ligands in MOF-5 thermally evaporate at 127–408 °C. Weight loss between 464 and 681 °C is also evident, which corresponds to the decomposition of MOF-5–CMC beads into inorganic substances. The above results indicate that the addition of MOF-5 effectively enhances the thermal stability of CMC, and the thermal stability of the new adsorbent is superior to those of both CMC and MOF-5.

### 3.3. XRD Analyses 

**Figure 3:**
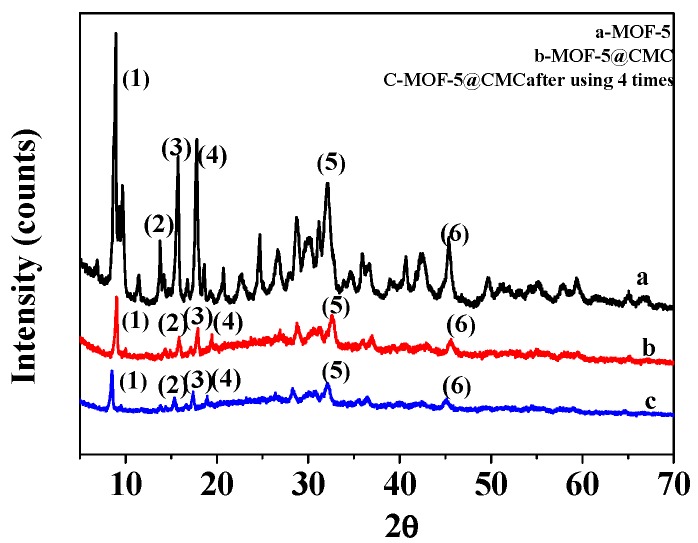
X-ray diffraction (XRD) patterns for (a) MOF-5, (b) MOF-5–CMC and (c) MOF-5–CMC after 4 uses.

Figure 3a,b display XRD patterns for MOF-5 and MOF-5–CMC, respectively. Six characteristic peaks are observed at 2 theta values of 8.9°, 13.8°, 15.8°, 17.9°, 32.0°, and 45.5°, indicating that the MOF-5 sample had been successfully synthesized [12]; the purity of the MOF-5 was high and its crystal structure was regular. MOF-5–CMC essentially exhibited the same characteristic peaks as MOF-5, which indicates that the structure of MOF-5 did not change as a result of being coated by CMC. However, there are some scattered peaks in the XRD pattern of MOF-5–CMC that indicate relatively poor MOF-5–CMC crystallinity. Figure 3c shows the XRD patterns of MOF-5–CMC after four uses; the results show that the crystalline form was retained during reuse.

### 3.4. XPS Analyses 

**Figure 4:**
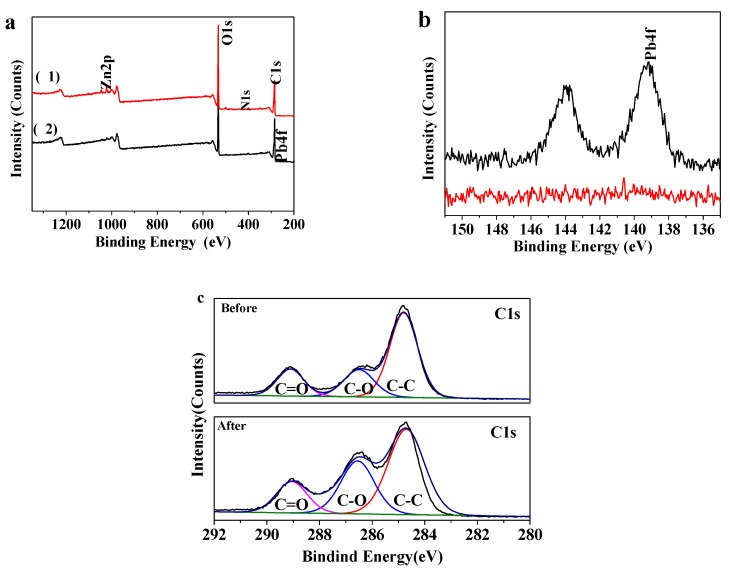
X-ray photoelectron spectroscopy (XPS) spectra of MOF-5–CMC: (**a**) survey spectra of (1) MOF-5–CMC and (2) Pb(II)-adsorbed MOF-5–CMC; (**b**) Pb 4f spectra of MOF-5–CMC before (lower) and after (upper) Pb(II) adsorption; C 1s spectra of MOF-5–CMC (**c**) before and after Pb(II) adsorption.

XPS spectra of MOF-5–CMC are displayed in Figure 4. Survey spectra before and after Pb(II) adsorption by MOF-5–CMC are shown in Figure 4a; a Zn 2p peak is observed at 1022.94 eV in the upper trace (1), while a small Pb 4f peak is observed in the lower trace (2), denoting that MOF-5–CMC adsorbed Pb successfully. The Pb 4f peak (139.25 eV) is more clearly seen in the enlarged spectrum (Figure 4b), revealing that lead-related chemicals are present on the surface of the MOF-5–CMC beads after Pb(II) was adsorbed. Figure 4c displays the C 1s spectra of the MOF-5–CMC beads prior to the attachment of Pb(II); peaks corresponding C–C, C–O, and C=O bonding appeared at 284.81, 286.2, and 289.1 eV, respectively. After adsorption of Pb(II), peaks corresponding C–C, C–O, and C=O were observed at 285.15, 286.9, and 289.5 eV; the shifts in these peaks are consistent with Pb(II) adsorption facilitated by the carboxyl groups in MOF-5–CMC [36,37].

### 3.5. FT-IR Analyses 

**Figure 5:**
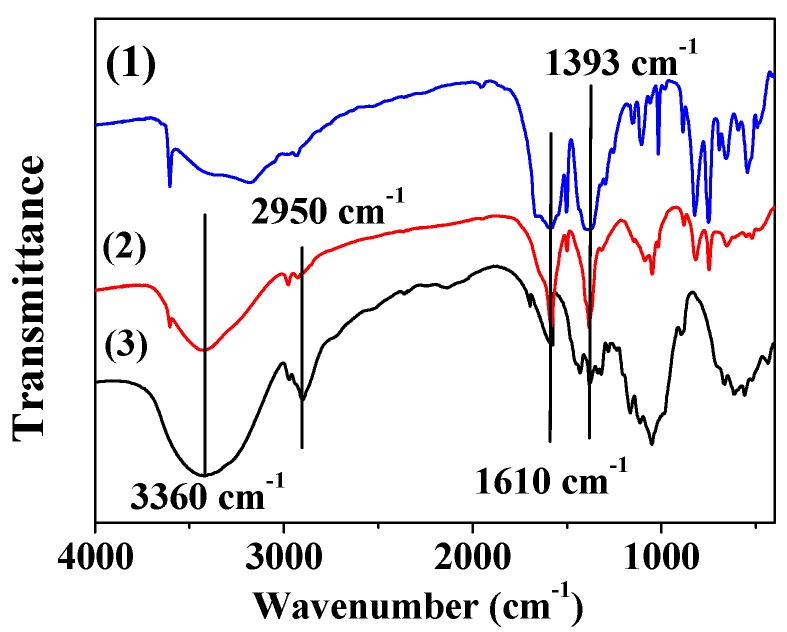
Fourier transform-infrared (FT-IR) spectra of (1) MOF-5, (2) MOF-5–CMC, and (3) CMC.

The FT-IR spectra of MOF-5, MOF-5–CMC, and CMC were acquired and are displayed in Figure 5. Bands at 1610 and 1393 cm^−1^ are due to asymmetric and symmetric COO-vibrations, respectively [38,39]. At 3360 cm^−1^, CMC and MOF-5–CMC exhibit -OH stretching bands, but this band is weak in the spectrum of MOF-5, which illustrates that the -OH stretching bands are mainly due to the presence of CMC [40,41]. In addition, CMC and MOF-5–CMC exhibited -CH_2_ stretching absorptions at 2950 cm^−1^, but the band corresponding to MOF-5–CMC was weaker than that of CMC [42]. Clearly, the carboxyl and hydroxyl groups play essential roles in the adsorption process.

### 3.6. Adsorption Experiments

We studied the effect of contact time between the adsorbent and the heavy-metal solution, adsorption temperature, pH, and the original concentration of the Pb(II), on adsorption behavior, for the purpose of determining the optimal adsorption conditions and to further understand the adsorption mechanism and properties of the adsorbent. In this study, 1 g of wet MOF-5–CMC beads corresponded to a dry weight of 45 mg. Wet CMC beads (1 g) corresponded to a dry weight of 24.21 mg.

#### 3.6.1. Effect of Contact Time

**Figure 6:**
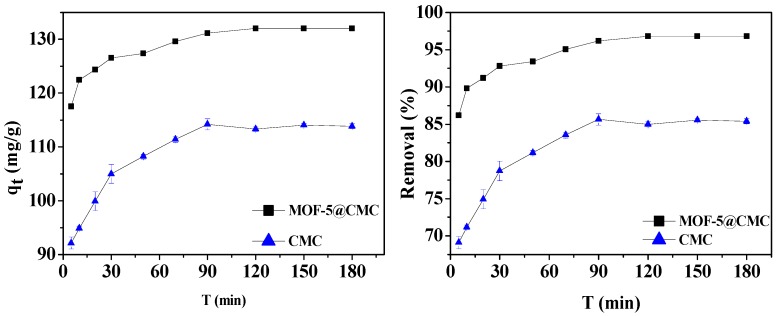
Adsorption capacity and removal rate as functions of contact time.

A 1-g sample of wet MOF-5–CMC beads was added to a 20 mL aliquot of a 300-mg/L Pb(II) solution. At the same time, 1 g CMC under the same conditions was selected as the control group. The optimum contact time for Pb(II) adsorption using the MOF-5–CMC beads was determined by examining adsorption capacity and removal rate as functions of time. Experimental observation times range between 5 and 360 min. Figure 6 reveals that the Pb(II)-adsorption capacity gradually increased between 5 and 120 min; it was 131.56 mg/g at 120 min, and no changes were observed with further increases in time. Therefore, we determined 120 min to be the optimal contact time, based on the absorption behavior of Pb(II), for use in further experiments.

#### 3.6.2. Effect of pH on Adsorption

**Figure 7:**
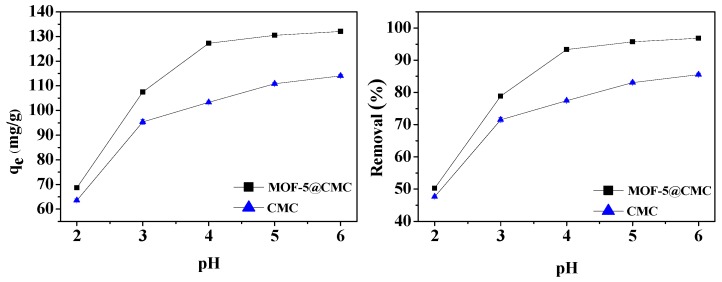
Adsorption capacity and removal rate as functions of pH.

Solution pH significantly influenced the adsorption capacity of Pb(II) by MOF-5–CMC. The pH was adjusted to lie between 2 to 6 using 0.1 M NaOH or 0.1 M HCl. Wet MOF-5–CMC beads (1 g) were added to 20 mL aliquots of a 300 mg/L Pb(II) solution with different pH. At the same time, CMC under the same conditions was selected as a blank control group. The relationship between pH and adsorption capacity is shown in Figure 7, which indicates that the ability to remove Pb(II) increased from 68.57 to 132.02 mg/g with increasing pH. At pH > 5, more than 95% Pb(II) removal was achieved. The carboxyl groups on the surface of the adsorbent are not easily ionized at lower pH, which is unfavorable for adsorption. The adsorption capacity and removal rate were stable at pH 5–6.

#### 3.6.3. Effect of Initial Solution Concentration 

**Figure 8:**
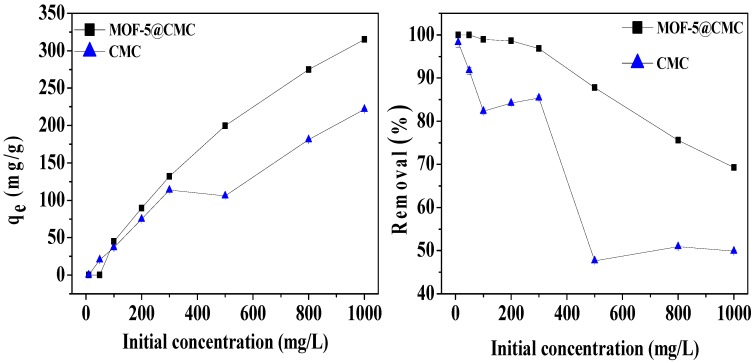
The action of initial Pb(II) concentration on adsorption capacity and removal rate.

We studied the effect of original concentration on adsorption capacity of the adsorbent with Pb(II) solutions at pH 6 and Pb(II) concentrations in the 10–1000 mg/L range. To ensure adsorption, each 20 mL Pb(II) solution was shaken for 120 min after the addition of 1 g of wet MOF-5–CMC beads at room temperature. As Figure 8 shows, the adsorption capacity for Pb(II) on the MOF-5–CMC beads increased from 44.98 to 315.05 mg/g as the original concentration of Pb(II) was increased from 10 to 1000 mg/L. This observation is explain as follows: when the concentration is low, the adsorption sites of the MOF-5–CMC beads do not reach saturation; the adsorption spots are gradually filled with increasing concentrations of Pb(II), resulting in an increase in adsorption capacity. Owing to the limited number adsorption sites on the surface of the adsorbent, the Pb(II)-removal rate by the MOF-5–CMC beads decreased gradually as the original concentration of Pb(II) was increased.

#### 3.6.4. Effect of Temperature on Adsorption

**Figure 9:**
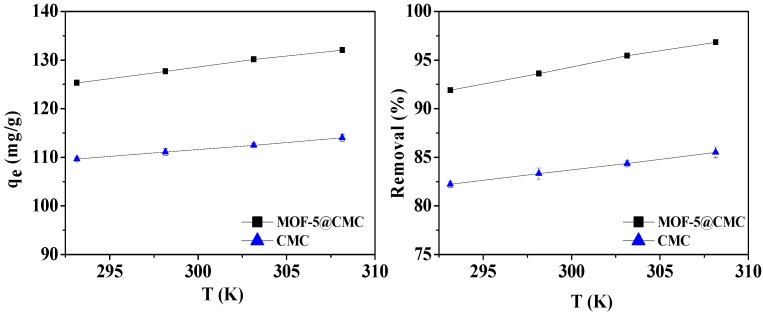
Adsorption capacity and removal rate as functions of temperature.

For the sake of studying the impact of temperature during the adsorption process, 20-mL aliquots of a 300-mg/L Pb(II) solution, in conical flasks, were treated at 293.15, 298.15, 303.15, and 308.15 K, with the adsorbent dose maintained at 1 g. At the same time, CMC was used as a control group. Figure 9 reveals that the adsorption capacity improves with increasing adsorption temperature. We conclude that higher temperatures are favorable for adsorbing Pb(II) onto the MOF-5–CMC beads, and the adsorption is endothermic.

#### 3.6.5. Effect of Adsorbent Dosage

**Figure 10:**
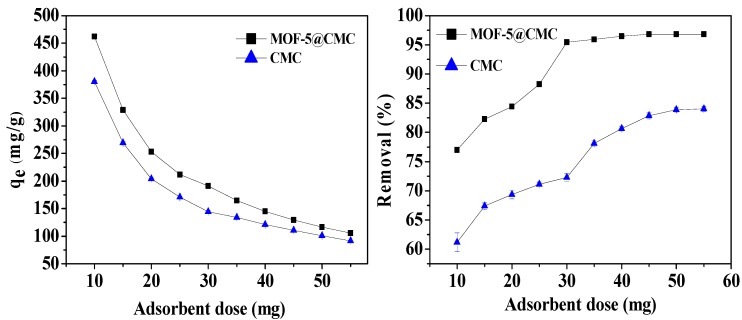
Adsorption capacity and removal rate as functions of adsorbent dose.

We also optimized the adsorbent dose; different adsorbent doses (10–55 mg) were added to 20 mL Pb(II) solution at a concentration of 300 mg/L at pH 6; the adsorption temperature was 298.15 K. According to Figure 10, the adsorption capacity decreased from 461.92 to 105.62 mg/g with increasing adsorbent dose. The Pb(II)-removal rate also increased from 76.9 to 96.82% and stabilized at a dose of 45 mg. Consequently, 45 mg was chosen as the adsorbent dose for further experiments.

### 3.7. Adsorption Mechanism

The mechanism for the adsorption of Pb(II) onto the MOF-5–CMC beads was investigated by kinetic modeling, Langmuir and Freundlich adsorption-isotherm analyses, and thermodynamics.

#### 3.7.1. Adsorption Kinetics

**Figure 11:**
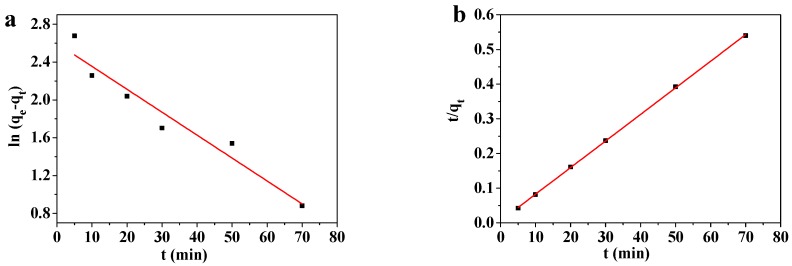
(**a**) Pseudo-first-order and (**b**) pseudo-second-order kinetics modeling of the adsorption of Pb(II) by MOF-5–CMC beads.

Solutions containing 10–1000 mg/L of Pb(II) were adsorbed at room temperature by 45 mg of the MOF-5–CMC beads and examined by pseudo-first-order and pseudo-second-order kinetic modeling. The results are showed in Table 1. Figure 11 shows that the obtained kinetic data matched the pseudo-first-order kinetic Equation (4) and the pseudo-second-order kinetic Equation (5):

(4) $$\ln\left(q_{e}-q_{t}\right)=\ln q_{e}-k_{1}t$$

(5) $$\frac{t}{q_{t}}=\frac{1}{k_{2}q_{e}^{2}}+\frac{t}{q_{e}}$$

Here *k*_1_ (1/min) is the pseudo-first-order kinetic rate constant and *k*_2_ (g/mg∙min) is the pseudo-second-order kinetic rate constant, with the other symbols as previously defined.

Figure 11 reveals that the Pb(II)-adsorption process involving the MOF-5–CMC adsorbent is better fitted by the pseudo-second-order kinetic model. The equilibrium adsorption capacity obtained by pseudo-second-order kinetics modeling is similar to that obtained experimentally. Therefore, we used the pseudo-second-order kinetic equation to explain the adsorption of Pb(II) onto the MOF-5–CMC adsorbent [43,44]; consequently, the Pb(II)-adsorption process involving MOF-5–CMC displays chemical-adsorption-like behavior.

#### 3.7.2. Adsorption Isotherm Modeling

**Figure 12:**
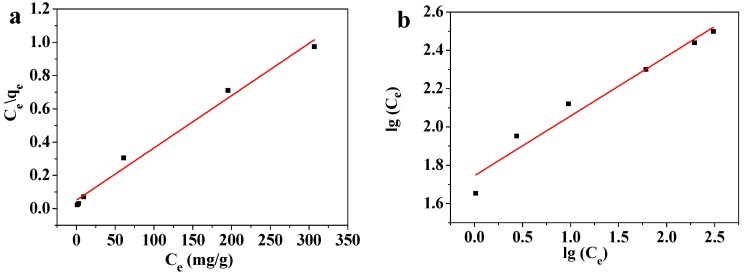
(**a**) Langmuir and (**b**) Freundlich isotherm modeling of the adsorption of Pb(II) on MOF-5–CMC beads.

Solutions containing 10–1000 mg/L of Pb(II) were adsorbed at room temperature by 45 mg of the MOF-5–CMC beads. The adsorption of Pb(II) by the adsorbent was fitted to the commonly used Langmuir adsorption-isotherm Equation (6) and the Freundlich adsorption-isotherm Equation (7), the results of which displayed in Figure 12; the isotherm model parameters obtained from these equations are listed in Table 2.

(6) $$\frac{C_{e}}{q_{e}}=\frac{l}{{bq}_{m}}+\frac{C_{e}}{q_{m}}$$

(7) $$\mathrm{lg}q_{e}=\mathrm{lg}K_{F}+\frac{l}{n}\mathrm{lg}C_{e}$$

Here *b* is the Langmuir constant, *K_F_* is the Freundlich constant that indicates the adsorption capacity, and *n* is the Freundlich constant that indicates adsorption strength, with the other symbols as previously defined.

In the Langmuir isotherm model, the dimensionless equilibrium constant *R_L_* is often used to judge whether or not adsorption is effective; *R_L_* can be calculated using Equation (8), the results of which are listed in Table 3:

(8) $$R_{L}=\frac{1}{1+bC_{0}}$$

where the symbols are as previously defined.

The Langmuir isotherm equation, with a high coefficient of determination, fitted the data well, which indicates that the adsorption of Pb(II) by MOF-5–CMC conforms to the law described by the Langmuir isotherm equation. These results reveal that the adsorbent mainly operates through monolayer adsorption [45]. The *q_m_* acquired from the fitted Langmuir-isotherm equation was 322.58 mg/g at room temperature. The Freundlich constant *n* is an important indicator of adsorption strength; in this experiment, *n* was determined to lie in the 1–10 range [46], while the Langmuir constant b was found to be positive (*b* > 0), which indicates that the theoretical saturation capacity of the MOF-5–CMC adsorbent is greater than the experimentally measured adsorption capacity [47]. Values of *R_L_* were between 0 and 1 (Table 3), indicating that this process is conducive to adsorption. In addition, *R_L_* was decreased with increasing Pb(II) concentration, indicating that higher concentrations favor the adsorption process [48]. We conclude that MOF-5–CMC is a good Pb(II) adsorbent.

#### 3.7.3. Adsorption Thermodynamics

Adsorption thermodynamics experiments were performed at different temperatures (293.15, 298.15, 303.15, and 308.15 K) in solutions containing 300 mg/L Pb(II) and 45 mg of adsorbent. The thermodynamic parameters Δ*H*, Δ*S*, and Δ*G* were determined by applying the thermodynamic formula:

(9) $$\Delta G=-RTln\frac{q_{e}}{C_{e}}=-RT\left(-\frac{\Delta H}{RT}+\frac{\Delta S}{R}\right)$$

The symbol *R* refers to the gas constant (8.314 J/mol·K) and *T* (K) is the absolute temperature of the solution.

As shown in Table 4, the *ΔG* for Pb(II) adsorption at concentration of 300 mg/L was negative at each temperature examined, indicating that Pb(II) adsorption by the MOF-5–CMC adsorbent occurs spontaneously. *ΔG* decreased with increasing temperature, which indicates that an appropriate elevated temperature will promote the adsorption process [49]. Δ*H* was positive, implying that the adsorption process is endothermic, which meets the requirements of the thermodynamic parameters [50]. Δ*S* was also positive, indicating that the adsorption process increases the entropy of the system [51]. In summary, an appropriate increase in temperature is conducive to adsorption.

### 3.8. Comparing the Pb(II)-Absorption Capacities of CMC, MOF-5, and CMC–MOF-5 Beads

**Figure 13:**
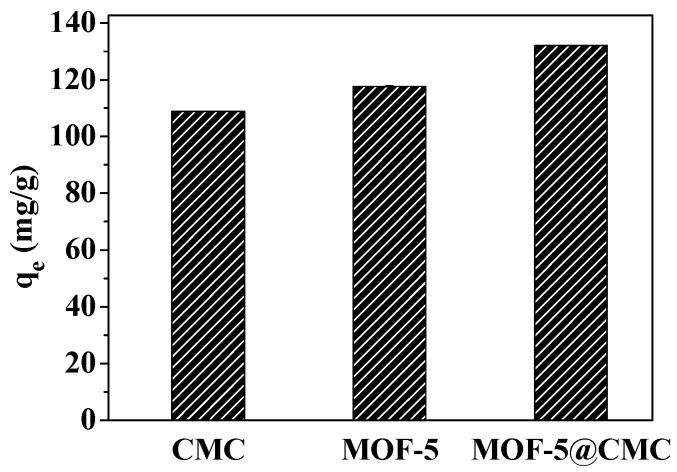
Pb(II) adsorption capacities of the adsorption materials in this study.

CMC and MOF-5 (45 mg each) and 1 g of wet MOF-5–CMC beads, were separately added to 20 mL aliquots of a 300-mg/L Pb(II) solution. The mixtures were treated for 120 min at room temperature and pH 6.0. As shown in Figure 13, the adsorption capacities of CMC, MOF-5, and MOF-5–CMC were 108.8, 117.67, and 132.04 mg/g at equilibrium. Clearly, the Pb(II)-adsorption capacity was significantly higher as a result of the CMC coating on the MOF-5 beads.

### 3.9. Regeneration Analysis

**Figure 14:**
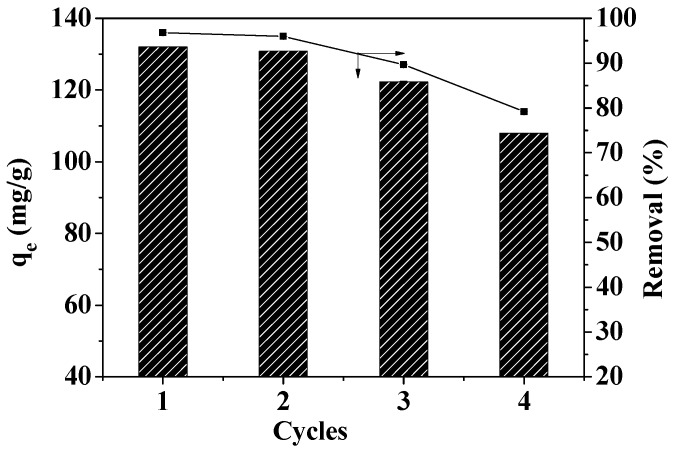
Reusability of MOF-5–CMC beads for the adsorption of Pb(II).

The ability to reuse materials greatly impacts the environment and the economy. In order to study reusability, the reuse efficiency of MOF-5–CMC was investigated. Figure 14 reveals that the Pb(II)-adsorption capacity of MOF-5–CMC decreased from 132.04 to 107.95 mg/g after four reuse cycles. However, the Pb(II)-removal rate was still 79% after four cycles of use, which indicates that MOF-5 seems to maintain its stability even after four adsorption cycles in aqueous solution, which somehow means that the formation of the MOF-5–CMC contributes to maintaining the stability of MOF-5. Therefore, MOF-5–CMC beads are a cost-effective and recyclable material.

## 4. Comparison of Adsorption Performance

The adsorption performance of MOF-5–CMC for Pb(II) was compared with those reported in previous papers. Compared with previous reports, the adsorbent prepared herein showed a marked increase in adsorption capacity. The results are shown in Table 5. MOF-5–CMC has the largest adsorption capacity (322.58 mg/g) and the adsorption time is 120 min, implying that MOF-5–CMC has good adsorption performance for Pb(II).

## 5. Conclusions

In summary, MOF-5–CMC beads are a new type of Pb(II) adsorbent with good adsorption properties. The influence of adsorption time, original concentration of Pb(II), pH, and adsorption temperature on the Pb(II)-adsorption process revealed that temperature has a slight influence on the adsorption process at pH 6. A maximum adsorption capacity of 132.13 mg/g was obtained under the optimal adsorption conditions. The adsorption process conforms to pseudo-second-order kinetics and is consistent with the Langmuir adsorption-isotherm model. Thermodynamics studies reveal that the adsorption process involving MOF-5–CMC beads is spontaneous and endothermic. We conclude that MOF-5–CMC is a convenient and cost-effective recyclable material. 

## Figures and Tables

**Table 1:** Pseudo-first-order and pseudo-second-order kinetic parameters for the adsorption of Pb(II) on MOF-5–CMC beads.

Concentration (mg/L)	Pseudo-First-Order Model			Pseudo-Second-Order Model		
	*q* *_e,exp_*	*k*_1_	*R* ^2^	*q* *_e,cal_*	*k*_2_	*R* ^2^
300	132.03	0.0242	0.945	133.64	0.0098	0.999

**Table 2:** Langmuir and Freundlich isotherm-model parameters for the adsorption of Pb(II) on MOF-5–CMC beads.

T (K)	Langmuir Isotherm			Freundlich Isotherm		
	*q_m_* (mg/g)	*b (L/mg)*	*R* ^2^	*K_F_*	*n*	*R* ^2^
293.15	322.58	0.061	0.988	1.75	3.22	0.961

**Table 3:** Langmuir equilibrium constants RL at various initial concentrations.

*C*_0_ (mg/L)	*R_L_*
100	0.14
200	0.075
300	0.052
500	0.032
800	0.02
1000	0.016

**Table 4:** Thermodynamic parameters for the adsorption of Pb(II) on MOF-5–CMC.

Concentration (mg/L)	Δ*G* (KJ/mol) *T* (K)				Δ*H* (KJ/mol)	Δ*S* (J/mol·K)
300	293.15 −3.99	298.15 −4.67	303.15 −5.69	308.15 −6.74	50.09	0.814

**Table 5:** Comparison of adsorption performance of MOF-5–CMC with previously reported adsorbents for Pb(II).

Adsorbents	*q_max_* (mg/g)	Adsorption Time	References
AS-ACI	133.3	150	[52]
Mg_2_Al-CO_3_-LDH	123	120	[53]
m-CS/PVA/CCNFs	175.4	200	[54]
ED-MIL-101	81.09	30	[55]
Zr-MOF	166.74	120	[56]
MOF-5	658.5	30	[57]
MOF-5–CMC	322.58	120	This work
